# Plasma levels of antimicrobial peptide LL-37 as a biomarker of sleep quality in patients with obstructive sleep apnea: A clinical observational study

**DOI:** 10.1371/journal.pone.0355855

**Published:** 2026-09-02

**Authors:** Takayuki Yamaguchi, Keisuke Hosokawa, Takenori Mineta, Rie Hosokawa, Shiho Odawara, Rie Chiba, Kazue Asanuma, Setsuko Toyomaki, Mitsuho Sato, Fumiaki Takahashi, Shigeru Sakurai, Kazuhiro Takahashi, Tsuguo Nishijima

**Affiliations:** 1 Division of Behavioral Sleep Medicine, Iwate Medical University School of Medicine, Shiwa, Japan; 2 Division of Information Sciences, Iwate Medical University, Shiwa, Japan; 3 MSD/ Seimindo Sleep Labo Systems, Morioka, Iwate, Japan; 4 Division of Internal Medicine, Yamaga Clinic, Takeda Healthcare Foundation, Aizu-Wakamatsu, Japan; Xuzhou Central Hospital, The Xuzhou School of Clinical Medicine of Nanjing Medical University, CHINA

## Abstract

**Objectives:**

Obstructive sleep apnea (OSA) is characterized by systemic inflammation, intermittent hypoxemia, and immune dysregulation and has been associated with increased susceptibility to viral infections. In this study, we aimed to evaluate plasma concentrations of the antimicrobial peptide cathelicidin LL-37 (pLL-37) in individuals with OSA.

**Methods:**

The associations between pLL-37 levels, demographic characteristics, and polysomnographic (PSG) parameters were assessed in 58 individuals with OSA and 20 healthy controls. Participants were stratified into two groups stratified by apnea–hypopnea index (AHI): AHI < 15 and AHI ≥ 15. Multiple regression analysis was conducted to identify independent predictors of pLL-37.

**Results:**

Significant negative correlations were observed between pLL-37 levels and age, percentage of Stage N1 sleep based on PSG data (%Stage N1), arousal index, AHI, and oxygen desaturation index. Conversely, significant positive correlations were identified between pLL-37 levels and %Stage N3, percentage of rapid eye movement sleep (%Stage REM), mean percentage of peripheral oxygen saturation (%SpO_2_ mean), and %SpO_2_ minimum. Mean pLL-37 level was significantly higher in the AHI < 15 group (75.3 ± 29.3 ng/L) than in the AHI ≥ 15 group (47.1 ± 18.3 ng/L). Multiple regression analysis identified %Stage N1 as an independent factor associated with pLL-37.

**Conclusions:**

pLL-37 levels were significantly reduced in individuals with severe OSA than in healthy controls. %Stage N1 was independently associated with pLL-37 levels, suggesting a decline with worsening oxygen desaturation. These findings suggest pLL-37 may serve as a biomarker for sleep quality and the severity of impaired oxygenation during sleep with implications for immune vulnerability and infection risk in OSA.

## 1. Introduction

Sleep-disordered breathing is associated with an increased risk of hypertension, cardiovascular disease [1], and mortality [2]. Obstructive sleep apnea (OSA) is a common disorder [3] that can be defined by an apnea–hypopnea index (AHI) of 5 events/h or higher on standard polysomnography (PSG); it is characterized by excessive daytime drowsiness and other symptoms [4]. OSA results from recurrent upper airway obstruction during sleep, leading to apneic or hypopneic episodes and sleep fragmentation. Obesity is an important risk factor for OSA [5]; however, even in individuals with low body mass index (BMI), craniofacial abnormalities such as micrognathia may increase the risk of OSA [6].

Regular sleep is crucial for maintaining immune function integrity and favoring homeostatic immune defense against microbial and inflammatory insults. The interaction between sleep and immunity is well established, with restorative sleep commonly recommended as a countermeasure against infectious diseases. Sleep, a fundamental physiological process occupying approximately one-third of the human lifespan, is essential for maintaining physical, psychological, and emotional well-being [7]. Both adequate sleep and a well-functioning immune system are necessary for maintaining good health; conversely, reduced total sleep time and poor sleep quality are associated with impaired immune function and increased susceptibility to illness [8,9]. Individuals with OSA are at increased risk of community-acquired pneumonia [10], influenza [11], and influenza-associated severe acute respiratory infections (SARI) [12]. Furthermore, comparative studies across diverse healthcare systems have demonstrated that OSA confers an approximately eightfold increased risk of coronavirus disease 2019 (COVID-19), and among infected individuals, OSA is associated with a twofold increase in hospitalization and progression to respiratory failure [13].

Recent advances have elucidated the molecular mechanisms by which pathogen recognition initiates inflammatory responses and promotes pathogen clearance, thereby improving our understanding of both innate and adaptive immunity. Cathelicidin LL-37, the only human cathelicidin-derived antimicrobial peptide, is generated through proteolytic cleavage of its precursor protein, human cationic antimicrobial protein-18 (hCAP18). LL-37 is a vitamin D-inducible peptide transcribed in response to the active form of vitamin D, 1,25-dihydroxycholecalciferol (1,25[OH]_2_D_3_), via activation of the vitamin D receptor [14]. It is primarily expressed by neutrophils, monocytes/macrophages, and epithelial cells and plays a central role in innate immunity. LL-37 exhibits broad-spectrum antimicrobial activity against bacteria, *Mycobacterium tuberculosis*, fungi, protozoa, and viruses and contributes to host defense against various pathogens. Low circulating levels of LL-37 have been associated with increased mortality from infectious diseases [15,16].

Beyond its antimicrobial activity, LL-37 functions as a multifunctional immunomodulatory molecule that regulates leukocyte chemotaxis, cytokine production, angiogenesis, and tissue repair, thereby contributing to both innate and adaptive immune responses. Furthermore, LL-37 expression is upregulated under inflammatory conditions and has been implicated in the pathogenesis of several inflammatory diseases. These biological properties suggest that alterations in circulating LL-37 levels in patients with OSA may reflect the systemic inflammatory state induced by intermittent hypoxia and sleep fragmentation. Moreover, given its immunomodulatory functions, LL-37 may not merely serve as a biomarker of inflammation but may also participate in disease pathophysiology as a mediator of inflammatory responses [15,17].

We hypothesized that patients with OSA may be susceptible to viral infections, potentially due to dysregulated activity of nucleic acid-sensing Toll-like receptors (TLRs) that detect viral mRNA during replication. Consequently, plasma LL-37 (pLL-37) concentrations may differ between patients with OSA and healthy controls. Specifically, we posit that pLL-37 concentrations are reduced in patients with OSA who did not have clinically active infection compared with levels in healthy controls or individuals with mild symptoms. In this study, we aimed to test this hypothesis by evaluating pLL-37 concentrations in individuals with OSA.

## 2. Participants and methods

### 2.1. Study design and participants

The participants were men who presented to Uchimaru Medical Center at Iwate Medical University of Morioka City, Iwate Prefecture, Japan, with complaints of snoring and suspected sleep apnea and who subsequently underwent overnight PSG. The cohort included 58 men diagnosed with OSA and 20 men classified as non-OSA based on PSG findings. Patients with a history of respiratory disease, atherosclerosis, immunodeficiency, or chronic infections were excluded from the study. In addition, all participants underwent comprehensive clinical evaluation, including detailed physical examination, complete blood count, serum biochemistry, and C-reactive protein (CRP) estimation. These assessments were performed to exclude the presence of other respiratory disorders, atherosclerotic disease, immunodeficiency, and any signs of acute or chronic infection at the time of enrollment. All patients were classified into two groups according to AHI: those with no to mild OSA (n = 40, AHI < 15 events/h), and those with moderate-to-severe OSA (n = 38, AHI ≥ 15 events/h). This study was conducted in accordance with the ethical principles outlined in the Declaration of Helsinki and received approval from the Ethics Committee of Iwate Medical University (Approval No. MH2024−141). As this was an observational study utilizing preexisting clinical data and archived biological samples, the protocol adhered to the provisions of the Ethical Guidelines for Life Science and Medical Research Involving Human Subjects. Specifically, the required disclosures concerning research conducted in the absence of informed consent were made publicly available. Opt-out materials were posted to allow participants or their legal representatives the opportunity to decline participation. Data from individuals who opted out were excluded from analysis and immediately destroyed. We accessed medical records, archived samples, and survey data for research purposes from August 25, 2025. We had accessed to information that could identify individual participants during or after data collection.

### 2.2. PSG study

All patients underwent overnight PSG using the Alice PDx system (Philips Respironics, Murrysville, PA) in a dedicated examination room to Uchimaru Medical Center at Iwate Medical University Hospital. Recordings commenced at 20:00 and concluded at 06:00 the following morning. Environmental and procedural conditions were standardized to minimize variability. Sleep data were scored and interpreted in accordance with version 2.5 of the Scoring Manual published by the American Academy of Sleep Medicine [18].

### 2.3. Collection of blood samples

On the morning following PSG performed for the diagnosis of OSA, venous blood was drawn from the antecubital region at 06:00, immediately upon the patient’s awakening. Blood was collected using a vacuum tube containing aprotinin. Plasma was immediately separated and cryopreserved at −60 °C until analysis.

### 2.4. Measurement of pLL-37 levels

Quantification of pLL-37 was performed using a high-sensitivity enzyme immunoassay (EIA) kit (rabbit; BMA Biomedicals AG, Augst, Switzerland). Prior to analysis, plasma samples were thawed at room temperature at approximately 20°C and purified using a C18 reverse-phase column. Based on a preliminary study, a 25-fold dilution of the purified eluate was used for final measurements.

### 2.5. Nasal continuous positive airway pressure (nCPAP)

Twenty out of the 58 patients with OSA were treated with nCPAP (REMstar Auto M series, Philips Respironics, Andover, MA). Twenty patients with OSA and AHI ≥ 30 events/h were selected for nCPAP treatment based on the application criteria for the Japanese social insurance system (AHI > 20 events/h). With informed consent, blood samples were obtained from these patients before and after the nCPAP treatment for a mean of approximately 3 months, and pLL-37 levels were measured. Sleep parameters before and after the treatment of nCPAP in these 20 patients are shown in Table 3. The automatic pressure adjustment mode (auto-CPAP) was used for the pressure setting for the initial 2 weeks of nCPAP treatment. An appropriate pressure was then determined based on the records from an Average Device Pressure <90% of the time of the treatment monitor (on-board memory) installed in the nCPAP device, and the pressure was adjusted during the following treatment period.

### 2.6. Statistical analysis

Statistical analyses were conducted using SPSS version 29.0 (IBM, Armonk, NY). Pearson’s product–moment correlation coefficient was used to examine the correlation between pLL-37 concentrations, PSG parameters, and demographic variables. Group comparisons of pLL-37 between the AHI < 15 and AHI ≥ 15 groups were analyzed using an unpaired *t*-*t*est. Comparisons of pLL-37 levels and clinical data before and after nCPAP treatment were done using paired Student’s t-tests. Data are presented as mean±standard error. Multiple regression analysis was also performed to analyze the factors that affected pLL-37. A two-sided p-value of 0.05 was considered to be statistically significant.

Sample size calculations were performed using G*Power version 3.1.9.6 [19,20]. For the Pearson correlation analysis, a minimum sample size of 26 was determined to be necessary to detect an effect size of 0.5 at a significance level of 5% and power of 80%. For the comparison between two unpaired groups, a sample size of at least 52 was required to detect an effect size of 0.8, significance level of 5% and power of 80%. Subsequent multiple regression analysis incorporated variables that showed significant associations with pLL-37 in the single regression analysis. For the multiple regression analysis, a sample size of at least 55 was calculated to be sufficient to detect an effect size of 0.15 at a significance level of 5% and power of 80%, assuming one tested predictor and a total of eight protectors included in the model.

## 3. Results

### 3.1. Analysis of patient demographic characteristics and sleep patterns

The study cohort comprised 58 men diagnosed with OSA (mean age: 52.2 ± 11.4 years, BMI: 23.9 ± 4.1 kg/m^2^) and 20 men diagnosed with non-OSA on PSG (mean age: 38.1 ± 14.8 years, BMI: 22.0 ± 1.7 kg/m^2^).

**Fig 1:**
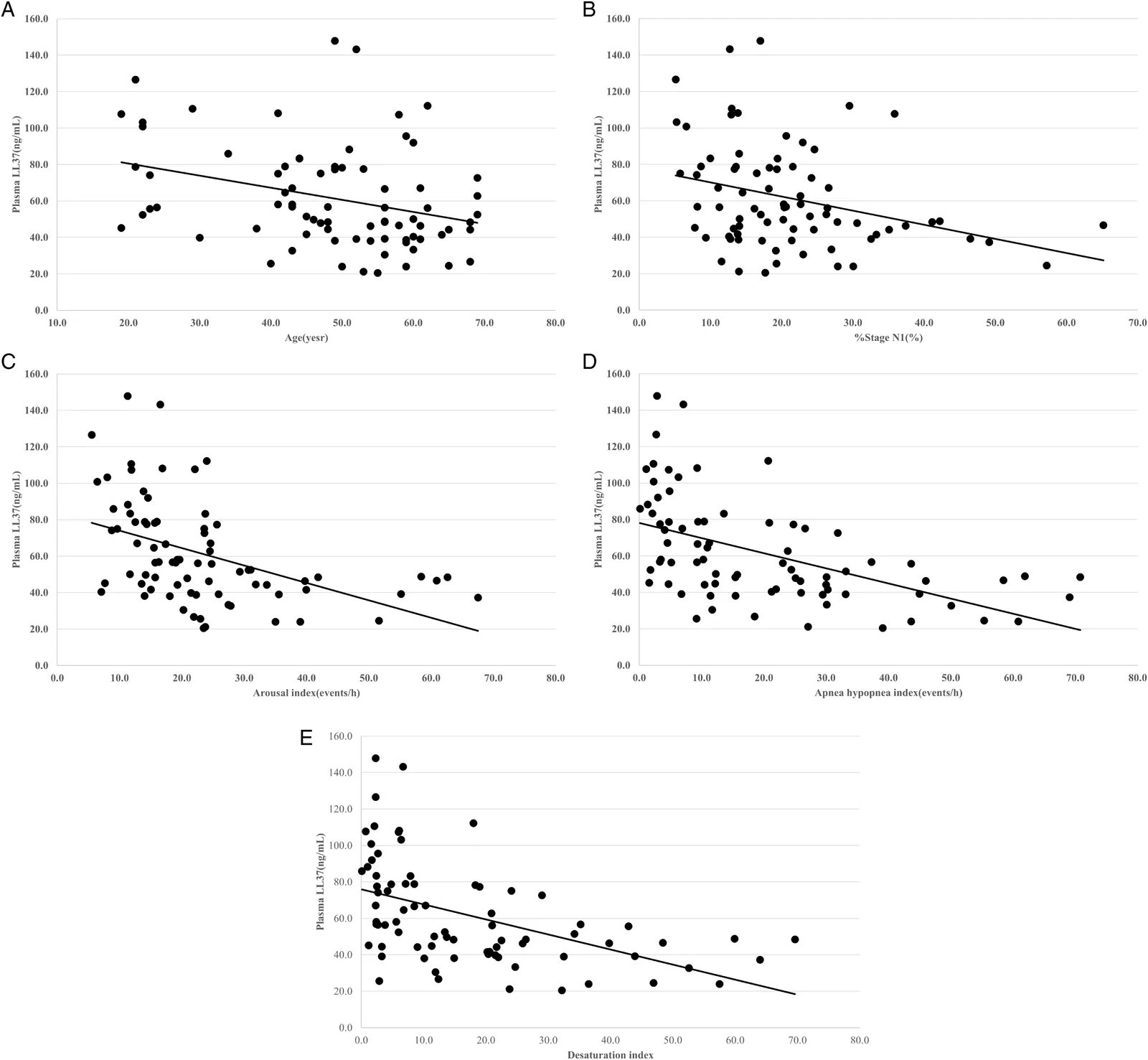
Correlation of pLL-37 concentration with clinical characteristics and polysomnography data. Age, (B)Percentage of Stage N1 sleep, (C)Arousal index, (D)Apnea hypopnea index, (E)Oxygen desaturation index (ODI).

The relationship of pLL-37 with demographic variables and PSG data is shown in Table 1. The mean pLL-37 across all participants was 61.5 ± 28.3 ng/L. Significant negative correlations were observed between pLL-37 and age (r = −0.323, p = 0.004; Fig 1A), percentage of Stage N1 sleep (%Stage N1) (r = −0.316, p = 0.005; Fig 1B), arousal index (r = −0.457, p < 0.0001; Fig 1C), AHI (r = −0.524, p < 0.0001; Fig 1D), and oxygen desaturation index (ODI) (r = −0.494, p < 0.0001; Fig 1E).

**Table 1:** Correlation of pLL-37 concentration with clinical characteristics and polysomnography data of the study cohort.

	LL-37		
**N = 78**		**r**	**p-value**
**Age (years)**	48.6 ± 13.7	−0.323	**0.004**
**Body mass index (kg/m**^**2**^)	23.4 ± 3.7	−0.154	0.117
**Total sleep time (min)**	455.4 ± 61.8	0.116	0.145
**Sleep efficiency (%)**	80.7 ± 10.4	0.174	0.127
**%Stage N1 (%)**	21.0 ± 11.5	−0.316	**0.005**
**%Stage N2 (%)**	52.6 ± 9.1	0.063	0.583
**%Stage N3 (%)**	8.4 ± 5.8	0.25	**0.027**
**%Stage REM (%)**	17.2 ± 4.9	0.258	**0.022**
**Arousal index (events/h)**	23.0 ± 13.5	−0.457	**<0.0001**
**Apnea hypopnea index (events/h)**	20.0 ± 17.8	−0.524	**<0.0001**
**Desaturation index (events/h)**	17.4 ± 16.9	−0.494	**<0.0001**
**%SpO**_**2**_ **mean (%)**	95.9 ± 1.1	0.336	**0.003**
**%SpO**_**2**_ **minimum (%)**	84.8 ± 8.2	0.239	**0.009**

pLL-37, antimicrobial peptide cathelicidin LL-37; N1, non-rapid eye movement (REM) sleep stage 1; N2, non-REM sleep stage 2; N3, non-REM sleep stage 3; SpO_2_, percentage of peripheral oxygen saturation. The data are presented as the mean ± standard error. A p-value ≤0.05 was considered statistically significant.

**Fig 2:**
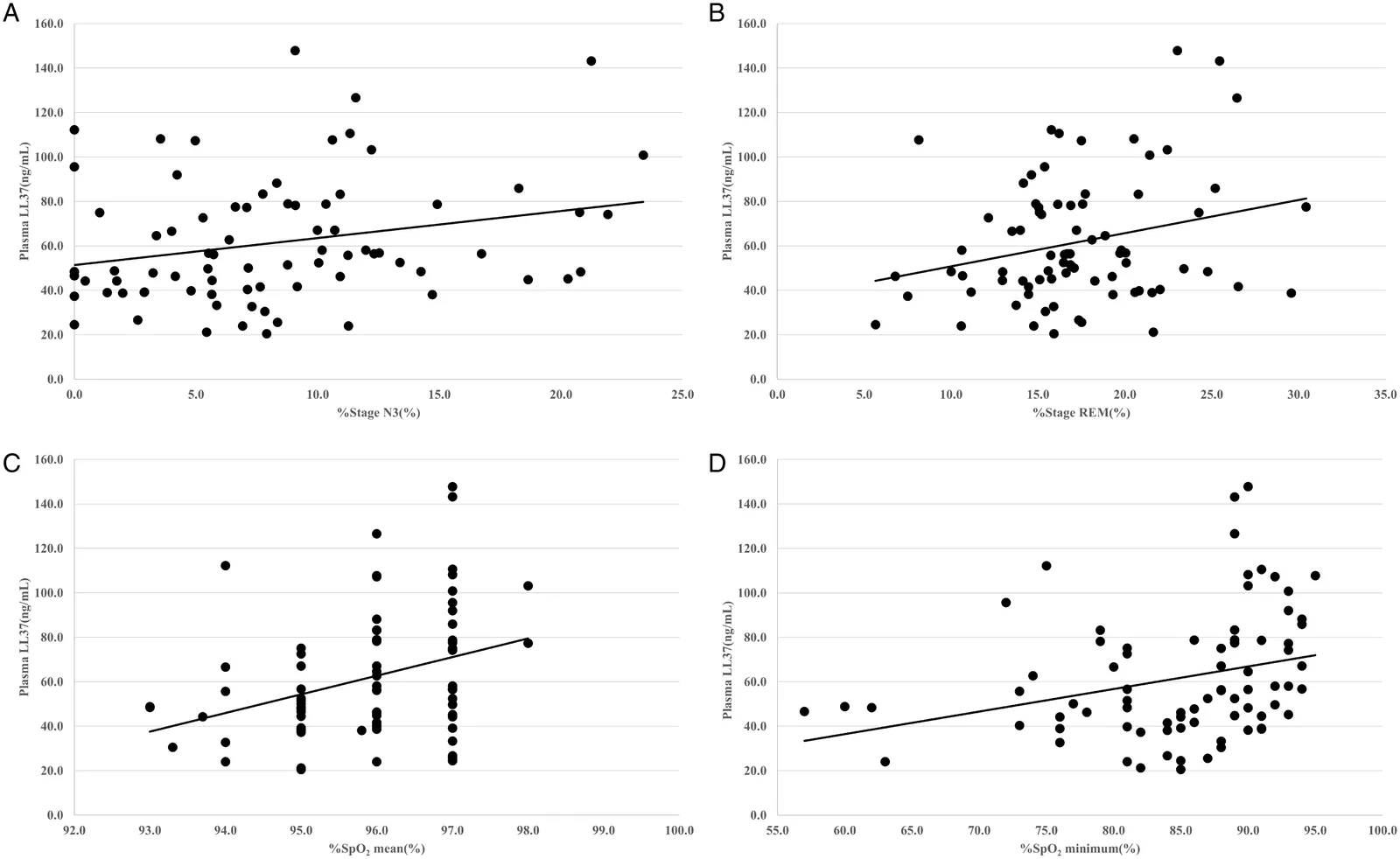
Correlation of pLL-37 concentration with polysomnography data. Percentage of Stage N3 sleep, (B)Percentage of Stage REM, (C) %SpO_2_ mean, (D) %SpO_2_ minimum.

Furthermore, significant positive correlations were found between pLL-37 and percentage of stage N3 sleep (%Stage N3) (r = 0.250, p = 0.027; Fig 2A), percentage of rapid eye movement sleep (%Stage REM) (r = 0.258, p = 0.022; Fig 2B), mean peripheral oxygen saturation (%SpO_2_ mean) (r = 0.336, p = 0.003; Fig 2C), and minimum SpO_2_ (%SpO_2_ minimum) (r = 0.239, p = 0.009; Fig 2D).

### 3.2. Comparison of variables between the AHI < 15 and AHI ≥ 15 groups

**Fig 3:**
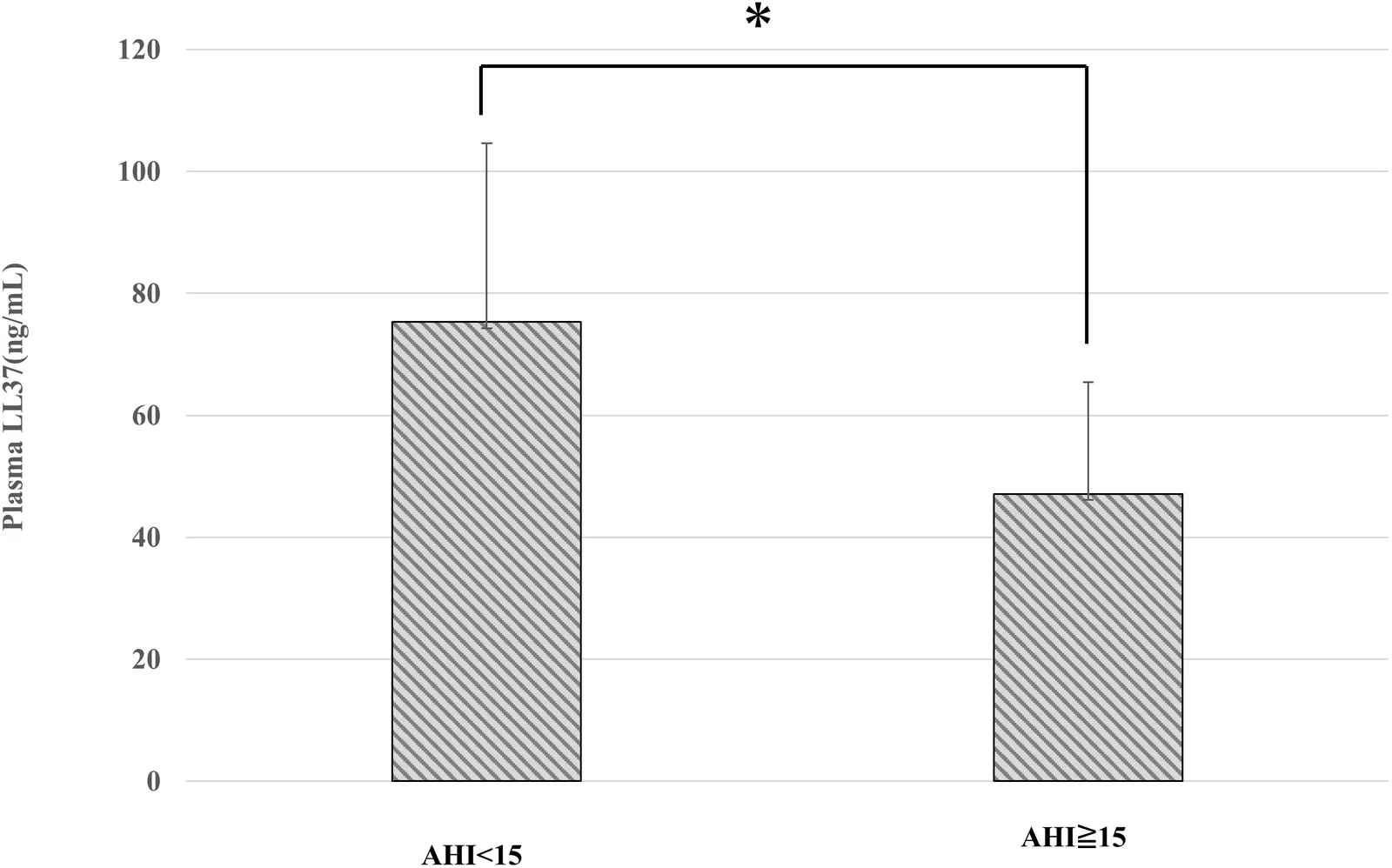
Comparison of pLL-37 levels between the AHI < 15 and AHI ≥ 15 groups.

The mean age of participants in the AHI < 15 group (42.4 ± 13.8 years) was significantly lower than that of participants in the AHI ≥ 15 group (48.6 ± 13.7 years; p < 0.001). With regard to PSG parameters, the AHI ≥ 15 group had significantly lower total sleep time (436.1 ± 56.5 vs. 473.7 ± 61.6 min; p = 0.006), sleep efficiency (77.4 ± 10.0% vs. 83.9 ± 9.9%; p = 0.005), %Stage N3 (6.4 ± 5.1 vs. 10.3 ± 5.9%; p = 0.003), mean SpO_2_ (95.3 ± 1.1% vs. 96.4 ± 0.9%; p < 0.001), and %SpO_2_ minimum (80.3 ± 8.7% vs. 89.0 ± 4.9%; p < 0.001), than did the AHI < 15 group. However, the AHI ≥ 15 group had significantly higher %Stage N1 (26.7 ± 12.9% vs. 15.7 ± 6.7%; p < 0.001), arousal index (30.6 ± 14.9 vs. 15.8 ± 6.2 events/h; p < 0.001), AHI (34.4 ± 15.2 vs. 6.3 ± 3.9 events/h; p < 0.001), and ODI (30.7 ± 15.2 vs. 4.9 ± 3.3 events/h; p < 0.001) compared to the AHI < 15 group. pLL-37 levels were significantly lower in the AHI ≥ 15 group (47.1 ± 18.3; p < 0.001) than in the AHI < 15 group (75.3 ± 29.3 ng/L) (Table 2, Fig 3).

**Table 2:** Comparison of clinical characteristics and polysomnography data between apnea–hypopnea index groups.

	AHI < 15 group (n = 40)	AHI ≥ 15 group (n = 38)	p-value
**Age (years)**	42.4 ± 13.8	48.6 ± 13.7	**<0.001**
**Body mass index (kg/m**^**2**^)	22.8 ± 2.7	24.0 ± 4.6	0.141
**Total sleep time (min)**	473.7 ± 61.6	436.1 ± 56.5	0.006
**Sleep efficiency (%)**	83.9 ± 9.9	77.4 ± 10.0	0.005
**%Stage N1 (%)**	15.7 ± 6.7	26.7 ± 12.9	**<0.001**
**%Stage N2 (%)**	54.4 ± 9.4	50.8 ± 8.6	0.075
**%Stage N3 (%)**	10.3 ± 5.9	6.4 ± 5.1	**0.003**
**%Stage REM (%)**	18.2 ± 4.4	16.2 ± 5.2	0.078
**Arousal index (events/h)**	15.8 ± 6.2	30.6 ± 14.9	**<0.001**
**Apnea hypopnea index (events/h)**	6.3 ± 3.9	34.4 ± 15.2	**<0.001**
**Desaturation index (events/h)**	4.9 ± 3.3	30.7 ± 15.2	**<0.001**
**%SpO**_**2**_ **mean (%)**	96.4 ± 0.9	95.3 ± 1.1	**<0.001**
**%SpO**_**2**_ **minimum (%)**	89.0 ± 4.9	80.3 ± 8.7	**<0.001**
**pLL-37 (ng/L)**	75.3 ± 29.3	47.1 ± 18.3	**<0.001**

N1, non-rapid eye movement (REM) sleep stage 1; N2, non-REM sleep stage 2; N3, non-REM sleep stage 3; pLL-37, antimicrobial peptide cathelicidin LL-37; SpO_2_, percentage of peripheral oxygen saturation. The data are presented as the mean ± standard error. A p-value ≤0.05 was considered statistically significant.

### 3.3. Changes in plasma pLL-37 concentrations after nCPAP treatment

As shown in Table 3, the sleep parameters including AHI and arousal index improved after treatment with nCPAP (duration of approximately 3 months) in the 20 patients with OSA.

**Table 3:** Comparison of sleep parameters before and after nasal continuous positive airway pressure (nCPAP) treatment in 20 patients with obstructive sleep apnea hypopnea syndrome.

N = 20	Before CPAP	After CPAP	p-value
**Total sleep time (min)**	435.0 ± 60.2	466.1 ± 45.5	0.019
**Sleep efficiency (%)**	76.9 ± 10.5	84.3 ± 7.0	0.004
**%Stage N1 (%)**	33.7 ± 13.3	19.3 ± 7.9	<0.001
**%Stage N2 (%)**	47.3 ± 9.1	51.7 ± 6.1	0.067
**%Stage N3 (%)**	5.1 ± 4.3	8.3 ± 4.1	0.002
**%Stage REM (%)**	13.9 ± 4.8	20.7 ± 5.0	0.002
**Arousal index (events/h)**	39.8 ± 14.8	16.4 ± 7.9	<0.001
**Apnea hypopnea index (events/h)**	45.0 ± 13.6	2.2 ± 1.2	<0.001
**Desaturation index (events/h)**	40.9 ± 14.3	1.7 ± 1.1	<0.001
**%SpO**_**2**_ **mean (%)**	94.9 ± 1.1	96.6 ± 0.8	<0.001
**%SpO**_**2**_ **minimum (%)**	76.8 ± 9.2	89.6 ± 5.8	<0.001
**pLL-37 (ng/L)**	41.8 ± 13.0	54.3 ± 28.3	0.052

pLL-37 concentrations tended to increase from 41.8 ± 13.0 to 54.3 ± 28.3 ng/mL (p = 0.052), but the difference was not significant.

### 3.4. Factors influencing pLL-37 concentration using multiple regression model

To identify factors associated with pLL-37, a multivariable regression analysis was conducted using age, %Stage N1, %Stage N3, %Stage REM, arousal index, AHI, ODI, mean SpO_2_, minimum SpO_2_, and BMI as independent variables, all of which showed significant correlations with pLL-37 in the univariate analysis (Tables 4 and 5). The analysis revealed that %Stage N1 (p = 0.040) and AHI (p = 0.038) were independently associated with pLL-37. Because multicollinearity was detected between AHI and ODI, ODI was excluded from the model, and the analysis was repeated. In the revised model, %Stage N1 (p = 0.045) and AHI (p = 0.014) remained significantly associated with pLL-37, suggesting that these variables are independently related to pLL-37 levels.

**Table 4:** Analysis of factors influencing pLL-37 concentrations using multiple regression model Full model.

	B	SE	Std. β	t value	p value	VIF
Variable	−179.876	333.923		-0.539	0.592	
Age (year)	−0.391	0.796	-0.052	-0.491	0.625	1.192
BMI (kg/m^2^)	−0.302	0.260	−0.147	−1.163	0.249	1.716
%Stage N1 (%)	1.140	0.545	0.465	2.091	**0.040**	5.320
%Stage N3 (%)	0.260	0.598	0.054	0.435	0.665	1.638
%Stage REM (%)	0.773	0.752	0.134	1.028	0.308	1.819
Arousal index (events/h)	−0.613	0.519	−0.293	−1.181	0.242	6.613
AHI (events/h)	−2.412	1.137	−1.523	−2.122	**0.038**	55.448
Desaturation index (events/h)	1.651	1.151	0.987	1.435	0.156	50.922
%SpO_2_ mean (%)	3.427	3.551	0.137	0.965	0.338	2.186
%SpO_2_ minimum (%)	−0.817	0.543	−0.237	−1.505	0.137	2.665

B, partial regression coefficient; SE, standard error; Std. β, standardized partial regression coefficient; VIF, variance inflation factor; N1, non-rapid eye movement (REM) sleep stage 1; N2, non-REM sleep stage 2; N3, non-REM sleep stage 3; Spo_2_, percentage of peripheral oxygen saturation.

**Table 5:** Analysis of factors influencing pLL-37 concentrations using multiple regression model Final model.

	B	SE	Std. β	t value	p value	VIF
Variable	−15.815	316.183		−0.050	0.960	
Age (year)	−0.353	0.802	−0.047	−0.440	0.661	1.191
BMI (kg/m^2^)	−0.407	0.252	−0.198	−1.617	0.111	1.582
%Stage N1 (%)	1.120	0.549	0.457	2.040	**0.045**	5.316
%Stage N3 (%)	0.243	0.602	0.050	0.403	0.688	1.637
%Stage REM (%)	0.800	0.758	0.138	1.055	0.295	1.818
Arousal index (events/h)	−0.681	0.521	−0.325	−1.307	0.196	6.559
AHI (events/h)	−0.854	0.340	−0.539	−2.513	**0.014**	4.882
%SpO_2_ mean (%)	1.773	3.385	0.071	0.524	0.602	1.955
%SpO_2_ minimum (%)	−0.840	0.547	−0.244	−1.537	0.129	2.663

B, partial regression coefficient; SE, standard error; Std. β, standardized partial regression coefficient; VIF, variance inflation factor; N1, non-rapid eye movement (REM) sleep stage 1; N2, non-REM sleep stage 2; N3, non-REM sleep stage 3; Spo_2_, percentage of peripheral oxygen saturation.

### 3.5. Cut-off value of pLL-37 for discriminating AHI < 15 and AHI > 15

To determine the optimal pLL-37 concentration for identifying patients with an AHI > 15, a receiver operating characteristic (ROC) curve was constructed, and the area under the curve (AUC) was calculated. The AUC was 0.804, indicating good discriminative performance. The maximum value of sensitivity − (1 − specificity) was 0.8. Based on logistic regression analysis, the optimal cut-off value of pLL-37 for diagnosing AHI > 15 was determined to be 56.4 ng/L.

The optimal cut-off value was determined by maximizing the Youden index. At this threshold, the sensitivity, specificity, and Youden index were 85%, 71%, and 0.56, respectively, indicating a moderate-to-good discriminatory ability for identifying patients with AHI > 15 events/h.

## 4. Discussion

Cathelicidin LL-37 exhibits broad-spectrum antimicrobial activity against a range of pathogens, including Gram-positive and Gram-negative bacteria, *M. tuberculosis*, protozoa, fungi, and viruses. In individuals with OSA who were free of clinically active infection, pLL-37 concentrations were lower in those with severe OSA, with levels inversely proportional to disease severity. The present study demonstrated that pLL-37 concentrations were significantly reduced in patients with OSA compared with those in healthy controls. Moreover, %Stage N1 was identified as a factor associated with pLL-37, suggesting a trend toward decreased LL-37 levels with increasing ODI. To our knowledge, this is the first report to show that pLL-37 concentrations are lower in individuals with OSA than in those without.

OSA is characterized by repeated upper airway collapse during sleep, resulting in apneic and hypopneic episodes. These events lead to sleep fragmentation, intermittent hypoxemia, and sympathetic hyperactivity, as evidenced by electroencephalographic findings. Such sleep-related stressors contribute to clinical manifestations including cardiac arrhythmias, vascular complications, and excessive daytime sleepiness. Notably, intermittent hypoxemia and sleep fragmentation significantly reduce sleep quality and may adversely affect immune function through central nervous system dysregulation and endocrine disturbances [21–23]. Reduced sleep efficiency and insufficient sleep duration are associated with susceptibility to respiratory viral infections [8], and poor sleep quality diminishes vaccine efficacy [24]. Epidemiological data indicate that individuals with OSA are more vulnerable to influenza [11] and community-acquired pneumonia [10]. Furthermore, OSA is associated with an approximately eightfold increased risk of COVID-19 infection compared to that in age-matched individuals receiving care in diverse healthcare settings. Among those infected with COVID-19, OSA has been correlated with higher rates of hospitalization and progression to respiratory failure [13]. Acute lower respiratory tract infections (LRTIs) in adults typically encompass bacterial and viral pneumonia and bronchitis [25], and OSA has been implicated in elevating the risk of SARI secondary to LRTI [25,26].

Although the precise mechanisms linking OSA to influenza-associated SARI remain unclear, etiological factors contributing to increased susceptibility likely involve multiple pathways. Mok et al. [27] inferred that the mechanisms underlying acute LRTI onset in patients with OSA may be attributed to three interrelated mechanisms: immune dysregulation, increased aspiration risk, and comorbid complications, which may act synergistically rather than independently. LL-37 binds directly to negatively charged nucleic acids, shielding them from enzymatic degradation by DNase and RNase. It enhances TLR3 signaling through direct interaction with double-stranded RNA [28,29] and forms complexes with single-stranded RNA [30] and single-stranded DNA to potentiate TLR7/TLR8 and TLR9 signaling, respectively. Conversely, LL-37:DNA complexes inhibit the formation of the AIM2 inflammasome via steric hindrance [31].

No prior studies have examined the relationship between OSA and LL-37. In this study, LL-37 was measured as a biomarker of innate immune defense in patients with OSA without active infection. The findings suggest that immune protection in OSA is modulated in a severity-dependent manner. Differences in LL-37 concentrations between healthy individuals and patients with OSA were further analyzed in relation to PSG-derived sleep indices. A significant inverse correlation was observed between LL-37 levels and %Stage N1, indicative of shallow sleep, while a significant positive correlation was found with %Stage N3, representing deep sleep. These results underscore a robust association between sleep quality and LL-37 concentration.

Additionally, a negative correlation was identified between LL-37 and ODI, a marker of respiratory impairment. This suggests that in OSA, both sleep fragmentation and intermittent hypoxemia may contribute to alterations in LL-37 levels. These changes may suppress inflammatory responses [31] mediated by LL-37, including activation of innate immune pathways such as TLR2 [32] and TLR4 [29,30], which recognize distinct bacterial cell wall components. pLL-37 may also participate in nucleic acid recognition mechanisms involving DNA, RNA, and polyribosomes, and its deficiency may disrupt the activation of nucleic acid-sensing TLRs.

Given that the study population was free of active infection and had no history of organic respiratory or atherosclerotic disease, and that confounding effects of active complications were minimized, it is likely that reduced LL-37 levels primarily reflect hypoxemia and sleep quality-based immune compromise.

We previously reported that plasma levels of soluble (pro)renin receptor [33] and plasma orexin-A [34] improve to the same level as in healthy individuals after approximately 3 months CPAP treatment. However, in this study, pLL-37 did not improve to the level of the AHI < 15 group 3 months after CPAP treatment. This may be due to the duration of treatment. Future studies are needed to determine how pLL-37 changes with long-term CPAP treatment.

### 4.1. Limitations

In this study, pLL-37 concentrations were independently associated with both the AHI and %Stage N1, a marker of sleep fragmentation and disrupted sleep architecture. However, because of the cross-sectional design of this study, causal relationships cannot be inferred from these associations.

OSA is characterized by intermittent hypoxia, oxidative stress, and systemic inflammation. Elevated pLL-37 levels may therefore reflect these pathophysiological processes and serve as a biomarker of OSA-related inflammatory activity. Conversely, LL-37 has been reported to participate in innate immune regulation, modulation of proinflammatory cytokine production, and tissue remodeling. These biological functions raise the possibility that LL-37 may also contribute to the development and progression of OSA-related pathophysiology. Nevertheless, current evidence is insufficient to determine whether LL-37 acts merely as a marker of disease activity or as a mediator of disease progression.

The association observed between pLL-37 and sleep architecture requires particular caution in interpretation. One possible explanation is that intermittent hypoxia and inflammatory responses associated with OSA promote sleep fragmentation and alterations in sleep architecture, leading to the observed relationship between pLL-37 and %Stage N1 sleep. However, no direct evidence demonstrates that LL-37 influences sleep architecture. Accordingly, our findings should be interpreted as demonstrating an association rather than a causal relationship.

Several limitations of this study should be acknowledged. First, the cross-sectional design precludes conclusions regarding causality. Second, this study focused solely on the quantification of circulating pLL-37 concentrations and did not assess the expression of related genes or mRNAs. Therefore, the biological mechanisms underlying the observed associations could not be clarified. Third, blood samples were collected only once in the early morning under fasting conditions. Because LL-37 concentrations may vary according to sampling conditions, including collection time and nutritional status, future studies should compare samples obtained from the same individuals at different timepoints, such as fasting morning and daytime measurements.

In addition, previous studies have reported a positive correlation between salivary LL-37 concentrations and age in children. The present study examined pLL-37 levels exclusively in adults with OSA; therefore, whether similar age-related associations exist in pediatric patients with OSA remains unknown and warrants further investigation.

Finally, LL-37 concentrations may be influenced by unmeasured factors unrelated to OSA, including unidentified comorbidities or other inflammatory conditions. Future longitudinal and mechanistic studies are needed to clarify the causal relationships among LL-37, OSA severity, sleep architecture, and systemic inflammation.

Overall, the available evidence supports a role for pLL-37 as a biomarker of OSA-related inflammation. However, its potential contribution as a biological mediator of OSA pathogenesis remains to be determined.

## 5. Conclusion

OSA was associated with reduced concentrations of pLL-37, potentially increasing susceptibility to infection. Furthermore, total sleep time and sleep quality were significantly correlated with LL-37 levels, highlighting the role of sleep in modulating innate immune defense. Our findings highlight the potential of pLL-37 as a biomarker for sleep quality and the severity of impaired oxygenation during sleep. Future studies are needed to clarify whether reduced LL-37 levels in OSA are a cause or consequence of immune dysfunction, particularly in OSA with complications such as active infection.

## Supporting information

S1 Data Sheet LL37 data sheet. (XLSX)
