# Supplementary material for: Plasma levels of antimicrobial peptide LL-37 as a biomarker of sleep quality in patients with obstructive sleep apnea: A clinical observational study
Source: PLoS One. 2026 Sep 2;21(9):e0355855. doi: 10.1371/journal.pone.0355855 (PMC13537548; doi:10.1371/journal.pone.0355855)
Supplement: Supplementary file 1 [file pone.0355855.s003.doc]

[24^th^ June 2026]

Dr. Helen Howard

*Staff Editor*

*PLOS One*

**Re: Manuscript reference PONE-D-25-66718R1**

Dear Dr. Howard,

Thank you very much for your kind email concerning our manuscript, entitled “Plasma levels of antimicrobial peptide LL-37 as a biomarker of sleep quality in patients with obstructive sleep apnea: a clinical observational study”.

We have carefully read your reviewers’ comments and revised our manuscript where possible. Point-by-point responses to reviewers can be found below, and all changes within the manuscript have been highlighted.

We believe that our manuscript has been greatly improved by these alterations, and hope that our manuscript now meets the high standards required for publication in *PLOS One.*

Thank you for your consideration. I look forward to hearing from you.

Sincerely,

Keisuke Hosokawa MD, PhD

Division of Sleep Medicine, Iwate Medical University School of Medicine, Morioka, Japan.

2-1-1 Idaidori, Yahaba Town, Iwate Prefecture

Phone: +81-19-651-5111 (ext. 4422)

Fax: +81-19-611-8062

E-mail: [khosokaw@iwate-med.ac.jp](mailto:khosokaw@iwate-med.ac.jp)

Response to the Reviewer

*Response:* We very much appreciate your kind and helpful comments. We have revised the manuscript according to your advice, and hope that you are satisfied with our responses.

Reviewer #3

Major Concerns Requiring Revision:

*Comment 1*. Sample size and power: The manuscript should explicitly state the sample size calculation, including the expected effect size, alpha level, and power. If formal power calculations were not performed prospectively, this must be acknowledged as a study limitation. The adequacy of sample size to detect meaningful correlations between LL-37 and PSG parameters needs justification.

*Response:* We have explained how we calculated the sample size in the Methods (2.6 Statistical analysis, lines 174–183):

“Sample size calculations were performed using G*Power version 3.1.9.6 [20, 21]. For the Pearson correlation analysis, a minimum sample size of 26 was determined to be necessary to detect an effect size of 0.5 at a significance level of 5% and power of 80%. For the comparison between two unpaired groups, a sample size of at least 52 was required to detect an effect size of 0.8, significance level of 5% and power of 80%. Subsequent multiple regression analysis incorporated variables that showed significant associations with pLL-37 in the single regression analysis. For the multiple regression analysis, a sample size of at least 55 was calculated to be sufficient to detect an effect size of 0.15 at a significance level of 5% and power of 80%, assuming one tested predictor and a total of eight protectors included in the model.”

*Comment 2*. Confounders not fully addressed: Plasma LL-37 levels are known to be influenced by vitamin D status, smoking, comorbid infections, BMI, and medications. The manuscript should provide a clear account of how these confounders were controlled or statistically adjusted. Specifically:

a. Vitamin D deficiency is strongly associated with both lower LL-37 levels and OSA severity was vitamin D status measured and controlled for?

b. BMI is a major confounder in OSA studies - were analyses adjusted for BMI?

c. Were participants on any medications known to affect inflammatory markers (e.g., statins, corticosteroids, CPAP users) excluded or separately analyzed?

*Response:* We conducted multiple linear regression analysis to identify factors associated with pLL-37. We have described the multiple linear regression analysis in the Results (lines 243–251):

“To identify factors associated with pLL-37, a multivariable regression analysis was conducted using age, %Stage N1, %Stage N3, %Stage REM, arousal index, AHI, ODI, mean SpO₂, minimum SpO₂, and BMI as independent variables, all of which showed significant correlations with pLL-37 in the univariate analysis. The analysis revealed that %Stage N1 (p = 0.040) and AHI (p = 0.038) were independently associated with pLL-37. Because multicollinearity was detected between AHI and ODI, ODI was excluded from the model, and the analysis was repeated. In the revised model, %Stage N1 (p = 0.045) and AHI (p = 0.014) remained significantly associated with pLL-37, suggesting that these variables are independently related to pLL-37 levels.”

a. We did not measure vitamin D levels in this study.
b. We did not adjust for BMI because no correlation was found between BMI and pLL-37. But in response to the reviewer's comment, we additionally included BMI as an independent variable in the multiple linear regression model to adjust for its potential confounding effect. The results were essentially unchanged after adjustment, confirming that BMI was not independently associated with pLL-37.
c. We excluded patients who used any medications known to affect inflammatory markers (such as statins and corticosteroids).

*Comment 3*. Causality and directionality: Given the cross-sectional observational design, the manuscript should avoid language that implies causality. Phrases suggesting that LL-37 changes "due to" OSA or hypoxic exposure should be revised to reflect the associational nature of the study.

*Response:* We agree with your opinion and have revised wording to reflect the associational nature of the study.

*Comment 4*. Biomarker validity: The authors should discuss the pre-analytical variables affecting LL-37 measurement (e.g., hemolysis, freeze-thaw cycles, time from collection to assay). Were samples collected at a standardized time (morning post-PSG)? Diurnal variation of LL-37 has not been well characterized and could introduce variability.

*Response:* In this study, on the morning following PSG performed for the diagnosis of OSA, we collected blood samples at 06:00, immediately upon the patient’s awakening. We have described the blood collection protocol as follows (lines 141–144):

“On the morning following PSG performed for the diagnosis of OSA, venous blood was drawn from the antecubital region at 06:00, immediately upon the patient’s awakening. Blood was collected using a vacuum tube containing aprotinin. Plasma was immediately separated and cryopreserved at −60 °C until analysis.”

*Comment 5*. ROC analysis: If the authors present ROC curve data for LL-37 as a diagnostic biomarker for OSA severity, the AUC, sensitivity, specificity, and optimal cut-off values should be clearly reported with 95% confidence intervals. The clinical utility of any proposed cut-off must be discussed in the context of existing OSA diagnosis (PSG remains the gold standard).

*Response:* To determine the optimal pLL-37 concentration for identifying patients with an AHI > 15, a receiver operating characteristic (ROC) curve was constructed, and the area under the curve (AUC) was calculated. We have described these results at lines 263–272:

“To determine the optimal pLL-37 concentration for identifying patients with an AHI > 15, a receiver operating characteristic (ROC) curve was constructed, and the area under the curve (AUC) was calculated. The AUC was 0.804, indicating good discriminative performance. The maximum value of sensitivity − (1 − specificity) was 0.8. Based on logistic regression analysis, the optimal cut-off value of pLL-37 for diagnosing AHI > 15 was determined to be 56.4 ng/L.

The optimal cut-off value was determined by maximizing the Youden index. At this threshold, the sensitivity, specificity, and Youden index were 85%, 71%, and 0.56, respectively, indicating a moderate-to-good discriminatory ability for identifying patients with AHI >15 events/h.”

*Comment 6*. Correlation vs. causation in discussion: The discussion should more carefully distinguish between LL-37 as a marker of systemic inflammation secondary to intermittent hypoxia versus a primary mediator of OSA-related pathology. The proposed mechanisms linking LL-37 to sleep architecture disruption are speculative and should be framed accordingly.

*Response:* We have added the following sentences to the limitations section (lines 342–361):

“In this study, pLL-37 concentrations were independently associated with both the AHI and %Stage N1, a marker of sleep fragmentation and disrupted sleep architecture. However, because of the cross-sectional design of this study, causal relationships cannot be inferred from these associations.

OSA is characterized by intermittent hypoxia, oxidative stress, and systemic inflammation. Elevated pLL-37 levels may therefore reflect these pathophysiological processes and serve as a biomarker of OSA-related inflammatory activity. Conversely, LL-37 has been reported to participate in innate immune regulation, modulation of proinflammatory cytokine production, and tissue remodeling. These biological functions raise the possibility that LL-37 may also contribute to the development and progression of OSA-related pathophysiology. Nevertheless, current evidence is insufficient to determine whether LL-37 acts merely as a marker of disease activity or as a mediator of disease progression.

The association observed between pLL-37 and sleep architecture requires particular caution in interpretation. One possible explanation is that intermittent hypoxia and inflammatory responses associated with OSA promote sleep fragmentation and alterations in sleep architecture, leading to the observed relationship between pLL-37 and %Stage N1 sleep. However, no direct evidence demonstrates that LL-37 influences sleep architecture. Accordingly, our findings should be interpreted as demonstrating an association rather than a causal relationship.”

Minor Concerns:

*Comment 1*. Figure quality: Ensure all figures meet PLOS ONE resolution requirements (300 DPI minimum) and that axis labels, legends, and statistical annotations are clearly readable.

*Response:* All figures in this article meet *PLOS One* resolution requirements (300 DPI minimum).

*Comment 2*. Tables: Baseline demographic and clinical characteristics (Table 1) should include p-values for between-group comparisons to confirm adequate matching or to justify covariate adjustment.

*Response:* We have shown the p-values in Table 1.

*Comment 3*. Reference list: Several foundational references on cathelicidin biology and its role in innate immunity (e.g., Zanetti 2004, Doss et al. 2010) should be cited to provide context for readers unfamiliar with LL-37 biology.

*Response:* We have added some papers to the reference list.

van Harten RM, van Woudenbergh E, van Dijk A, Haagsman HP. Cathelicidins: immunomodulatory antimicrobials. Vaccines (Basel). 2018;6: 63. doi: 10.3390/vaccines6030063.

Dürr UH, Sudheendra US, Ramamoorthy A. LL-37, the only human member of the cathelicidin family of antimicrobial peptides. Biochim Biophys Acta. 2006;1758: 1408-1425. doi: 10.1016/j.bbamem.2006.03.030

*Comment 4*. Abbreviations: Ensure all abbreviations are defined at first use and that a consistent abbreviation style is used throughout.

*Response:* We have double-checked all abbreviations, which are presented in a consistent style.

*Comment 5*. Limitations section: The limitations section should explicitly acknowledge: (a) cross-sectional design precluding causal inference, (b) potential unmeasured confounders (vitamin D, infections at time of sampling), (c) single-center design limiting generalizability, and (d) the fact that PSG was performed once without accounting for night-to-night variability in sleep architecture.

*Response:* We have added information to the discussion (lines 362–383).

“Several limitations of this study should be acknowledged. First, the cross-sectional design precludes conclusions regarding causality. Second, this study focused solely on the quantification of circulating pLL-37 concentrations and did not assess the expression of related genes or mRNAs. Therefore, the biological mechanisms underlying the observed associations could not be clarified. Third, blood samples were collected only once in the early morning under fasting conditions. Because LL-37 concentrations may vary according to sampling conditions, including collection time and nutritional status, future studies should compare samples obtained from the same individuals at different timepoints, such as fasting morning and daytime measurements.

In addition, previous studies have reported a positive correlation between salivary LL-37 concentrations and age in children. The present study examined pLL-37 levels exclusively in adults with OSA; therefore, whether similar age-related associations exist in pediatric patients with OSA remains unknown and warrants further investigation.

Finally, LL-37 concentrations may be influenced by unmeasured factors unrelated to OSA, including unidentified comorbidities or other inflammatory conditions. Future longitudinal and mechanistic studies are needed to clarify the causal relationships among LL-37, OSA severity, sleep architecture, and systemic inflammation.

Overall, the available evidence supports a role for pLL-37 as a biomarker of OSA-related inflammation. However, its potential contribution as a biological mediator of OSA pathogenesis remains to be determined.”

*Comment 6*. CPAP/treatment status: Were any OSA patients already on CPAP therapy? If so, this would significantly confound LL-37 levels, as CPAP treatment reduces systemic inflammation in OSA. This must be clarified.

*Response:* None of the study participants had received CPAP therapy before the start of the study.

Additional revisions made by the authors.

*Revision 1*. We have corrected typos in the text.

*Revision 2*. In this study, the cohort included “58” men diagnosed with OSA and “38” men with moderate-to-severe OSA. In lines 111, 120, and 187, we described “59” men diagnosed with OSA and “39” men with moderate-to-severe OSA. This was corrected accordingly.

*Revision 3*. Typos in lines 195, 207, 214, and 215 were corrected.

*Revision 4*. In lines 221–222, the sentence was revised to “pLL-37 levels were significantly lower in the AHI≥15 group (47.1±18.3; p<0.001) than in the AHI<15 group (75.3±29.3 ng/L)”.

*Revision 5*. We have added four papers to our reference list, which increased the number of references to 17.
