# Supplementary material for: Plasma levels of antimicrobial peptide LL-37 as a biomarker of sleep quality in patients with obstructive sleep apnea: A clinical observational study
Source: PLoS One. 2026 Sep 2;21(9):e0355855. doi: 10.1371/journal.pone.0355855 (PMC13537548; doi:10.1371/journal.pone.0355855)
Supplement: Peer review history

# Peer review: Decision Letter 0

**DOI:** 10.1371/journal.pone.0355855.r001

**Authors:** Helen Howard

25 Feb 2026

Plasma levels of antimicrobial peptide LL-37 as a biomarker of sleep quality in patients with obstructive sleep apnea: a clinical observational study

PLOS One

Dear Dr. Hosokawa,

Thank you for submitting your manuscript to PLOS ONE. After careful consideration, we feel that it has merit but does not fully meet PLOS ONE’s publication criteria as it currently stands. Therefore, we invite you to submit a revised version of the manuscript that addresses the points raised during the review process.

Could you please revise the manuscript to carefully address the concerns raised?

Please submit your revised manuscript by Apr 11 2026 11:59PM. If you will need more time than this to complete your revisions, please reply to this message or contact the journal office at plosone@plos.org. When you're ready to submit your revision, log on to https://www.editorialmanager.com/pone/ and select the 'Submissions Needing Revision' folder to locate your manuscript file.

Please include the following items when submitting your revised manuscript:

- A letter that responds to each point raised by the academic editor and reviewer(s). You should upload this letter as a separate file labeled 'Response to Reviewers'.
- A marked-up copy of your manuscript that highlights changes made to the original version. You should upload this as a separate file labeled 'Revised Manuscript with Track Changes'.
- An unmarked version of your revised paper without tracked changes. You should upload this as a separate file labeled 'Manuscript'.

If you would like to make changes to your financial disclosure, please include your updated statement in your cover letter. Guidelines for resubmitting your figure files are available below the reviewer comments at the end of this letter.

If applicable, we recommend that you deposit your laboratory protocols in protocols.io to enhance the reproducibility of your results. Protocols.io assigns your protocol its own identifier (DOI) so that it can be cited independently in the future. For instructions see: https://journals.plos.org/plosone/s/submission-guidelines#loc-laboratory-protocols. Additionally, PLOS ONE offers an option for publishing peer-reviewed Lab Protocol articles, which describe protocols hosted on protocols.io. Read more information on sharing protocols at https://plos.org/protocols?utm_medium=editorial-email&utm_source=authorletters&utm_campaign=protocols.

We look forward to receiving your revised manuscript.

Kind regards,

Helen Howard

Staff Editor

PLOS One

Journal Requirements:

When submitting your revision, we need you to address these additional requirements.

1. Please ensure that your manuscript meets PLOS ONE's style requirements, including those for file naming. The PLOS ONE style templates can be found at

https://journals.plos.org/plosone/s/file?id=wjVg/PLOSOne_formatting_sample_main_body.pdf and https://journals.plos.org/plosone/s/file?id=ba62/PLOSOne_formatting_sample_title_authors_affiliations.pdf

2. Please provide additional details regarding participant consent. In the ethics statement in the Methods and online submission information, please ensure that you have specified what type you obtained (for instance, written or verbal, and if verbal, how it was documented and witnessed). If your study included minors, state whether you obtained consent from parents or guardians. If the need for consent was waived by the ethics committee, please include this information.

Once you have amended this/these statement(s) in the Methods section of the manuscript, please add the same text to the “Ethics Statement” field of the submission form (via “Edit Submission”).

For additional information about PLOS ONE ethical requirements for human subjects research, please refer to http://journals.plos.org/plosone/s/submission-guidelines#loc-human-subjects-research.

3. We note that you have indicated that there are restrictions to data sharing for this study. For studies involving human research participant data or other sensitive data, we encourage authors to share de-identified or anonymized data. However, when data cannot be publicly shared for ethical reasons, we allow authors to make their data sets available upon request. For information on unacceptable data access restrictions, please see http://journals.plos.org/plosone/s/data-availability#loc-unacceptable-data-access-restrictions.

Before we proceed with your manuscript, please address the following prompts:

a) If there are ethical or legal restrictions on sharing a de-identified data set, please explain them in detail (e.g., data contain potentially identifying or sensitive patient information, data are owned by a third-party organization, etc.) and who has imposed them (e.g., a Research Ethics Committee or Institutional Review Board, etc.). Please also provide contact information for a data access committee, ethics committee, or other institutional body to which data requests may be sent.

b) If there are no restrictions, please upload the minimal anonymized data set necessary to replicate your study findings to a stable, public repository and provide us with the relevant URLs, DOIs, or accession numbers. Please see http://www.bmj.com/content/340/bmj.c181.long for guidelines on how to de-identify and prepare clinical data for publication. For a list of recommended repositories, please see https://journals.plos.org/plosone/s/recommended-repositories. You also have the option of uploading the data as Supporting Information files, but we would recommend depositing data directly to a data repository if possible.

Please update your Data Availability statement in the submission form accordingly.

4. Thank you for stating the following in the Competing Interests section:

[The authors have declared that no competing interests exist.].

We note that one or more of the authors are employed by a commercial company: MSD/ Seimindo Sleep Labo Systems

1. Please provide an amended Funding Statement declaring this commercial affiliation, as well as a statement regarding the Role of Funders in your study. If the funding organization did not play a role in the study design, data collection and analysis, decision to publish, or preparation of the manuscript and only provided financial support in the form of authors' salaries and/or research materials, please review your statements relating to the author contributions, and ensure you have specifically and accurately indicated the role(s) that these authors had in your study. You can update author roles in the Author Contributions section of the online submission form.

Please also include the following statement within your amended Funding Statement.

“The funder provided support in the form of salaries for authors [insert relevant initials], but did not have any additional role in the study design, data collection and analysis, decision to publish, or preparation of the manuscript. The specific roles of these authors are articulated in the ‘author contributions’ section.”

If your commercial affiliation did play a role in your study, please state and explain this role within your updated Funding Statement.

2. Please also provide an updated Competing Interests Statement declaring this commercial affiliation along with any other relevant declarations relating to employment, consultancy, patents, products in development, or marketed products, etc.

Within your Competing Interests Statement, please confirm that this commercial affiliation does not alter your adherence to all PLOS ONE policies on sharing data and materials by including the following statement: "This does not alter our adherence to PLOS ONE policies on sharing data and materials.” (as detailed online in our guide for authors http://journals.plos.org/plosone/s/competing-interests) . If this adherence statement is not accurate and there are restrictions on sharing of data and/or materials, please state these. Please note that we cannot proceed with consideration of your article until this information has been declared.

Please include both an updated Funding Statement and Competing Interests Statement in your cover letter. We will change the online submission form on your behalf.

5. We note that you have included the phrase “data not shown” in your manuscript. Unfortunately, this does not meet our data sharing requirements. PLOS does not permit references to inaccessible data. We require that authors provide all relevant data within the paper, Supporting Information files, or in an acceptable, public repository. Please add a citation to support this phrase or upload the data that corresponds with these findings to a stable repository (such as Figshare or Dryad) and provide and URLs, DOIs, or accession numbers that may be used to access these data. Or, if the data are not a core part of the research being presented in your study, we ask that you remove the phrase that refers to these data.

6. Your ethics statement should only appear in the Methods section of your manuscript. If your ethics statement is written in any section besides the Methods, please delete it from any other section.

7. Please include a separate caption for each figure in your manuscript.

If the reviewer comments include a recommendation to cite specific previously published works, please review and evaluate these publications to determine whether they are relevant and should be cited. There is no requirement to cite these works unless the editor has indicated otherwise.

[Note: HTML markup is below. Please do not edit.]

Reviewers' comments:

Reviewer's Responses to Questions

**Comments to the Author**

1. Is the manuscript technically sound, and do the data support the conclusions?

Reviewer #1: Yes

**********

2. Has the statistical analysis been performed appropriately and rigorously?

Reviewer #1: Yes

**********

3. Have the authors made all data underlying the findings in their manuscript fully available?

The PLOS Data policy

Reviewer #1: No

**********

4. Is the manuscript presented in an intelligible fashion and written in standard English?

Reviewer #1: Yes

**********

Reviewer #1: The submitted article addresses LL-37 as a biomarker in OSA, linking sleep-disordered breathing to innate immunity and potential infection risk. The authors describe a plausible immunological role of LL-37 that could be linked to an OSA-related hypoxia and/or fragmented sleep. This was supported by significant correlations with %Stage N1, %Stage N3, and the oxygen desaturation index. Although the presented document has potential clinical relevance, particularly regarding infection susceptibility, it shows some key gaps:

∙ When describing the study population, in addition to the PSG-based AHI classification criteria, the authors should also specify participants' treatment status (e.g., treatment-naïve, untreated, or under CPAP therapy).

∙ The authors suggest that hypoxemia and sleep quality drive LL-37 levels but provide no intervention data to support this causality. If feasible, the authors could examine changes in LL-37 levels after using CPAP or following an OSA management plan; this would further support the proposed causal relationship between hypoxemia and LL-37 levels.

∙ Line 273-274: “diminished pLL-37 levels may impair inflammasome inhibition, which is primarily responsible for susceptibility to viral infection” is more speculative without direct supporting data from this study or previous studies.

∙ Line 297-299: Suggesting the consideration of vaccination strategies based on LL-37 levels in the current report and in the absence of interventional or prospective infection evidence is premature. Further evidence/studies required to put forward such a strategy.

∙ Data presentation should be improved, and better quality plots/figures should be included.

∙ Sample type of LL-37 and timing of collection (i.e. time of day of collection; fasting status and diurnal consideration) appears to have a detrimental effect on the outcome. Considering this fact with LL-37, how would it serve as a potential biomarker for OSA?

∙ Discuss generalizability to diverse populations. Would LL-37 be a good marker for patients with OSA, both paediatric and adult?

∙ What’s next, or how can these results be taken further for validation or exploration? Suggest a potential future plan e.g. longitudinal tracking of LL-37 with CPAP initiation, or potential in vitro studies to test how LL-37 variations might influence mucosal immunity in the context of hypoxia/fragmentation.

In general the English requires some improvement

**********

what does this mean?). If published, this will include your full peer review and any attached files.

If you choose “no”, your identity will remain anonymous but your review may still be made public.

**Do you want your identity to be public for this peer review?**  For information about this choice, including consent withdrawal, please see our Privacy Policy

Reviewer #1: No

**********

[NOTE: If reviewer comments were submitted as an attachment file, they will be attached to this email and accessible via the submission site. Please log into your account, locate the manuscript record, and check for the action link "View Attachments". If this link does not appear, there are no attachment files.]

To ensure your figures meet our technical requirements, please review our figure guidelines: https://journals.plos.org/plosone/s/figures

You may also use PLOS’s free figure tool, NAAS, to help you prepare publication quality figures: https://journals.plos.org/plosone/s/figures#loc-tools-for-figure-preparation.

NAAS will assess whether your figures meet our technical requirements by comparing each figure against our figure specifications.

# Author comment: Author response to Decision Letter 1

**DOI:** 10.1371/journal.pone.0355855.r002

10 Apr 2026

Response to the Reviewer

Response: We very much appreciate your kind and helpful comments. We have revised the manuscript according to your advice, and hope that you are satisfied with our responses.

Reviewer #1

Comment 1. When describing the study population, in addition to the PSG-based AHI classification criteria, the authors should also specify participants' treatment status (e.g., treatment-naïve, untreated, or under CPAP therapy).

Response: Twenty out of the 58 patients with obstructive sleep apnea (OSA) were treated with nasal continuous positive airway pressure (nCPAP). Twenty patients with OSA and apnea–hypopnea index (AHI) ≥30 events/h were selected for nCPAP treatment based on the application criteria for the Japanese social insurance system (AHI > 20 events/h).

We have added a description of CPAP treatment to the Methods section:

“Twenty out of the 58 patients with OSA were treated with nCPAP (REMstar Auto M series, Philips Respironics, Andover, MA). Twenty patients with OSA and AHI ≥30 events/h were selected for nCPAP treatment based on the application criteria for the Japanese social insurance system (AHI > 20 events/h). With informed consent, blood samples were obtained from these patients before and after the nCPAP treatment for a mean of approximately 3 months, and pLL-37 levels were measured. Sleep parameters before and after the treatment of nCPAP in these 20 patients are shown in Table 3. The automatic pressure adjustment mode (auto-CPAP) was used for the pressure setting for the initial 2 weeks of nCPAP treatment. An appropriate pressure was then determined based on the records　from the Average Device Pressure <90% of the time of the treatment monitor (on-board memory) installed in the nCPAP device, and the pressure was adjusted during the following treatment period.”

Comment 2. The authors suggest that hypoxemia and sleep quality drive LL-37 levels but provide no intervention data to support this causality. If feasible, the authors could examine changes in LL-37 levels after using CPAP or following an OSA management plan; this would further support the proposed causal relationship between hypoxemia and LL-37 levels.

Response: We measured the pLL-37 before and after CPAP treatment. pLL-37 concentrations tended to increase from 41.8 ± 13.0 to 54.3 ± 28.3 ng/mL (p=0.052), but the difference was not significant. We have added sentences about pLL-37 before and after CPAP therapy to the Results section:

“As shown in Table 3, the sleep parameters including AHI and ArI improved after treatment with nCPAP (duration of approximately 3 months) in the 20 patients with OSA.

pLL-37 concentrations tended to increase from 41.8 ± 13.0 to 54.3 ± 28.3 ng/mL(p=0.052), but the difference was not significant.”

Comment 3. Line 273-274: “diminished pLL-37 levels may impair inflammasome inhibition, which is primarily responsible for susceptibility to viral infection” is more speculative without direct supporting data from this study or previous studies.

Response: We agree with your opinion; we have removed this sentence because we lacked supporting data.

Comment 4. Line 297-299: Suggesting the consideration of vaccination strategies based on LL-37 levels in the current report and in the absence of interventional or prospective infection evidence is premature. Further evidence/studies required to put forward such a strategy.

Response: We agree with your opinion; we have removed this sentence because we lacked supporting data.

Comment 5. Data presentation should be improved, and better quality plots/figures should be included.

Response: We have improved the quality of plots/figures.

Comment 6. Sample type of LL-37 and timing of collection (i.e. time of day of collection; fasting status and diurnal consideration) appears to have a detrimental effect on the outcome. Considering this fact with LL-37, how would it serve as a potential biomarker for OSA?

Response: In this study, we collected samples in the early morning when patients were fasting. Therefore, we could not investigate the effects of timing of collection. We have added the following sentences to the Discussion section:

“Additionally, in this study, samples were collected in the early morning when patients were fasting. Thus, the timing and circumstances of sample collection (time of collection, whether the patient had eaten, etc.) may have affected pLL-37 levels. Therefore, it is considered necessary to collect blood from the same patients both in the early morning when they are fasting and during the daytime and compare the results.”

Comment 7. Discuss generalizability to diverse populations. Would LL-37 be a good marker for patients with OSA, both paediatric and adult?

Response: In this study, we did not include pediatric patients. We have added the following sentences to the Discussion section:

“Furthermore, studies on LL-37 concentration in saliva in children have reported a positive correlation with age. This study examined pLL-37 in adults with OSA. Future research should investigate whether similar trends exist in pediatric patients with OSA.”

Comment 8. What’s next, or how can these results be taken further for validation or exploration? Suggest a potential future plan e.g. longitudinal tracking of LL-37 with CPAP initiation, or potential in vitro studies to test how LL-37 variations might influence mucosal immunity in the context of hypoxia/fragmentation.

Response: pLL-37 concentrations tended increased from 41.8 ± 13.0 to 54.3 ± 28.3 ng/mL (p=0.052), but the difference was not significant. On the other hand, we did not investigate effects of age and timing of sample collection. In future research, we think that the effects of age and timing of sample collection should be investigated to make pLL-37 a more common marker.

Response to journal requirement

Comment 3. We note that you have indicated that there are restrictions to data sharing for this study. For studies involving human research participant data or other sensitive data, we encourage authors to share de-identified or anonymized data. However, when data cannot be publicly shared for ethical reasons, we allow authors to make their data sets available upon request.

b) If there are no restrictions, please upload the minimal anonymized data set necessary to replicate your study findings to a stable, public repository and provide us with the relevant URLs, DOIs, or accession numbers. Please see http://www.bmj.com/content/340/bmj.c181.long for guidelines on how to de-identify and prepare clinical data for publication. For a list of recommended repositories, please see https://journals.plos.org/plosone/s/recommended-repositories. You also have the option of uploading the data as Supporting Information files, but we would recommend depositing data directly to a data repository if possible.

Please update your Data Availability statement in the submission form accordingly.

Response: We upload the dataset as a Supporting Information files.

Comment 4. Thank you for stating the following in the Competing Interests section:

[The authors have declared that no competing interests exist.].

We note that one or more of the authors are employed by a commercial company: MSD/ Seimindo Sleep Labo Systems

Response: The authors received no specific funding for this work from MSD/Seimindo Sleep Labo Systems.

Attachment Submitted filename: Responce_to_Reviewers_PLOS_ONE_LL-37h4.doc

# Peer review: Decision Letter 1

**DOI:** 10.1371/journal.pone.0355855.r003

**Authors:** Yongzhong Guo

26 May 2026

Dear Dr. Hosokawa,

Thank you for submitting your manuscript to PLOS ONE. After careful consideration, we feel that it has merit but does not fully meet PLOS ONE’s publication criteria as it currently stands. Therefore, we invite you to submit a revised version of the manuscript that addresses the points raised during the review process.

Please submit your revised manuscript by Jul 10 2026 11:59PM. If you will need more time than this to complete your revisions, please reply to this message or contact the journal office at plosone@plos.org. When you're ready to submit your revision, log on to https://www.editorialmanager.com/pone/ and select the 'Submissions Needing Revision' folder to locate your manuscript file.

- A letter that responds to each point raised by the academic editor and reviewer(s). You should upload this letter as a separate file labeled 'Response to Reviewers'.
- A marked-up copy of your manuscript that highlights changes made to the original version. You should upload this as a separate file labeled 'Revised Manuscript with Track Changes'.
- An unmarked version of your revised paper without tracked changes. You should upload this as a separate file labeled 'Manuscript'.

If you would like to make changes to your financial disclosure, please include your updated statement in your cover letter. Guidelines for resubmitting your figure files are available below the reviewer comments at the end of this letter.

If applicable, we recommend that you deposit your laboratory protocols in protocols.io to enhance the reproducibility of your results. Protocols.io assigns your protocol its own identifier (DOI) so that it can be cited independently in the future. For instructions see: https://journals.plos.org/plosone/s/submission-guidelines#loc-laboratory-protocols. Additionally, PLOS ONE offers an option for publishing peer-reviewed Lab Protocol articles, which describe protocols hosted on protocols.io. Read more information on sharing protocols at https://plos.org/protocols?utm_medium=editorial-email&utm_source=authorletters&utm_campaign=protocols.

As the corresponding author, your ORCID iD is verified in the submission system and will appear in the published article. PLOS supports the use of ORCID, and we encourage all coauthors to register for an ORCID iD and use it as well. Please encourage your coauthors to verify their ORCID iD within the submission system before final acceptance, as unverified ORCID iDs will not appear in the published article. *Only*  the individual author can complete the verification step; PLOS staff *cannot*  verify ORCID iDs on behalf of authors.

We look forward to receiving your revised manuscript.

Kind regards,

Yongzhong Guo, Ph.D

Academic Editor

PLOS One

Journal Requirements:

If the reviewer comments include a recommendation to cite specific previously published works, please review and evaluate these publications to determine whether they are relevant and should be cited. There is no requirement to cite these works unless the editor has indicated otherwise.

[Note: HTML markup is below. Please do not edit.]

Reviewers' comments:

Reviewer's Responses to Questions

**Comments to the Author**

Reviewer #1: All comments have been addressed

Reviewer #2: All comments have been addressed

Reviewer #3: (No Response)

**********

2. Is the manuscript technically sound, and do the data support the conclusions?

Reviewer #1: Yes

Reviewer #2: Yes

Reviewer #3: Partly

**********

3. Has the statistical analysis been performed appropriately and rigorously?

Reviewer #1: Yes

Reviewer #2: Yes

Reviewer #3: Yes

**********

4. Have the authors made all data underlying the findings in their manuscript fully available?

The PLOS Data policy

Reviewer #1: Yes

Reviewer #2: Yes

Reviewer #3: Yes

**********

5. Is the manuscript presented in an intelligible fashion and written in standard English?

Reviewer #1: Yes

Reviewer #2: Yes

Reviewer #3: Yes

**********

Reviewer #1: The authors have thoroughly and satisfactory addressed all of my previous comments in their rebuttal. I am satisfied with the revision and recommend acceptance.

Reviewer #2: Thank You for submitting the revised version and addressing all the questions.I would suggest future research to study the diurnal variations of PPL37 in untreated and treated OSA and whether such variations increase the risk of infection.

Reviewer #3: REVIEW OF MANUSCRIPT PONE-D-25-66718R1

Title: Plasma levels of antimicrobial peptide LL-37 as a biomarker of sleep quality in patients with obstructive sleep apnea: a clinical observational study

Overall Assessment:

This revised manuscript investigates plasma LL-37 (cathelicidin) levels as a potential biomarker of sleep quality in OSA patients. The topic is clinically relevant given the well-established link between OSA, intermittent hypoxia, systemic inflammation, and innate immune dysregulation. The authors have made efforts to address prior reviewer comments. However, several important concerns remain that require attention before the manuscript can be accepted.

Strengths:

1. The study addresses a genuinely novel question regarding the role of cathelicidin LL-37 in OSA pathophysiology, extending prior work on antimicrobial peptides in respiratory disease (e.g., COPD) to sleep-disordered breathing.

2. The use of polysomnography (PSG) for OSA diagnosis and sleep quality assessment adds rigor compared to studies relying solely on questionnaires or oximetry.

3. The observational design with appropriate comparator groups (OSA patients vs. controls) is methodologically appropriate for biomarker discovery.

4. The inclusion of correlational analyses between LL-37 levels and PSG parameters (AHI, oxygen desaturation index, minimum SpO2, sleep efficiency, etc.) is commendable.

Major Concerns Requiring Revision:

1. Sample size and power: The manuscript should explicitly state the sample size calculation, including the expected effect size, alpha level, and power. If formal power calculations were not performed prospectively, this must be acknowledged as a study limitation. The adequacy of sample size to detect meaningful correlations between LL-37 and PSG parameters needs justification.

2. Confounders not fully addressed: Plasma LL-37 levels are known to be influenced by vitamin D status, smoking, comorbid infections, BMI, and medications. The manuscript should provide a clear account of how these confounders were controlled or statistically adjusted. Specifically:

a. Vitamin D deficiency is strongly associated with both lower LL-37 levels and OSA severity - was vitamin D status measured and controlled for?

b. BMI is a major confounder in OSA studies - were analyses adjusted for BMI?

c. Were participants on any medications known to affect inflammatory markers (e.g., statins, corticosteroids, CPAP users) excluded or separately analyzed?

3. Causality and directionality: Given the cross-sectional observational design, the manuscript should avoid language that implies causality. Phrases suggesting that LL-37 changes "due to" OSA or hypoxic exposure should be revised to reflect the associational nature of the study.

4. Biomarker validity: The authors should discuss the pre-analytical variables affecting LL-37 measurement (e.g., hemolysis, freeze-thaw cycles, time from collection to assay). Were samples collected at a standardized time (morning post-PSG)? Diurnal variation of LL-37 has not been well characterized and could introduce variability.

5. ROC analysis: If the authors present ROC curve data for LL-37 as a diagnostic biomarker for OSA severity, the AUC, sensitivity, specificity, and optimal cut-off values should be clearly reported with 95% confidence intervals. The clinical utility of any proposed cut-off must be discussed in the context of existing OSA diagnosis (PSG remains the gold standard).

6. Correlation vs. causation in discussion: The discussion should more carefully distinguish between LL-37 as a marker of systemic inflammation secondary to intermittent hypoxia versus a primary mediator of OSA-related pathology. The proposed mechanisms linking LL-37 to sleep architecture disruption are speculative and should be framed accordingly.

Minor Concerns:

1. Figure quality: Ensure all figures meet PLOS ONE resolution requirements (300 DPI minimum) and that axis labels, legends, and statistical annotations are clearly readable.

2. Tables: Baseline demographic and clinical characteristics (Table 1) should include p-values for between-group comparisons to confirm adequate matching or to justify covariate adjustment.

3. Reference list: Several foundational references on cathelicidin biology and its role in innate immunity (e.g., Zanetti 2004, Doss et al. 2010) should be cited to provide context for readers unfamiliar with LL-37 biology.

4. Abbreviations: Ensure all abbreviations are defined at first use and that a consistent abbreviation style is used throughout.

5. Limitations section: The limitations section should explicitly acknowledge: (a) cross-sectional design precluding causal inference, (b) potential unmeasured confounders (vitamin D, infections at time of sampling), (c) single-center design limiting generalizability, and (d) the fact that PSG was performed once without accounting for night-to-night variability in sleep architecture.

6. CPAP/treatment status: Were any OSA patients already on CPAP therapy? If so, this would significantly confound LL-37 levels, as CPAP treatment reduces systemic inflammation in OSA. This must be clarified.

Conclusion:

The manuscript presents an interesting exploratory observation about LL-37 in OSA patients. With appropriate revisions addressing sample size justification, confounder control, clearer causal framing, and more robust discussion of biomarker validity, the manuscript could make a meaningful contribution to the literature. I recommend Minor Revision with a request for the authors to provide a detailed response to these concerns.

**********

what does this mean?). If published, this will include your full peer review and any attached files.

If you choose “no”, your identity will remain anonymous but your review may still be made public.

**Do you want your identity to be public for this peer review?**  For information about this choice, including consent withdrawal, please see our Privacy Policy

Reviewer #1: No

Reviewer #2: **Yes:**  Shais Jallu

Reviewer #3: No

**********

[NOTE: If reviewer comments were submitted as an attachment file, they will be attached to this email and accessible via the submission site. Please log into your account, locate the manuscript record, and check for the action link "View Attachments". If this link does not appear, there are no attachment files.]

To ensure your figures meet our technical requirements, please review our figure guidelines: https://journals.plos.org/plosone/s/figures

You may also use PLOS’s free figure tool, NAAS, to help you prepare publication quality figures: https://journals.plos.org/plosone/s/figures#loc-tools-for-figure-preparation.

NAAS will assess whether your figures meet our technical requirements by comparing each figure against our figure specifications.

# Author comment: Author response to Decision Letter 2

**DOI:** 10.1371/journal.pone.0355855.r004

26 Jun 2026

Response to the Reviewer

Response: We very much appreciate your kind and helpful comments. We have revised the manuscript according to your advice, and hope that you are satisfied with our responses.

Reviewer #3

Major Concerns Requiring Revision:

Comment 1. Sample size and power: The manuscript should explicitly state the sample size calculation, including the expected effect size, alpha level, and power. If formal power calculations were not performed prospectively, this must be acknowledged as a study limitation. The adequacy of sample size to detect meaningful correlations between LL-37 and PSG parameters needs justification.

Response: We have explained how we calculated the sample size in the Methods (2.6 Statistical analysis, lines 174–183):

“Sample size calculations were performed using G*Power version 3.1.9.6 [20, 21]. For the Pearson correlation analysis, a minimum sample size of 26 was determined to be necessary to detect an effect size of 0.5 at a significance level of 5% and power of 80%. For the comparison between two unpaired groups, a sample size of at least 52 was required to detect an effect size of 0.8, significance level of 5% and power of 80%. Subsequent multiple regression analysis incorporated variables that showed significant associations with pLL-37 in the single regression analysis. For the multiple regression analysis, a sample size of at least 55 was calculated to be sufficient to detect an effect size of 0.15 at a significance level of 5% and power of 80%, assuming one tested predictor and a total of eight protectors included in the model.”

Comment 2. Confounders not fully addressed: Plasma LL-37 levels are known to be influenced by vitamin D status, smoking, comorbid infections, BMI, and medications. The manuscript should provide a clear account of how these confounders were controlled or statistically adjusted. Specifically:

a. Vitamin D deficiency is strongly associated with both lower LL-37 levels and OSA severity was vitamin D status measured and controlled for?

b. BMI is a major confounder in OSA studies - were analyses adjusted for BMI?

c. Were participants on any medications known to affect inflammatory markers (e.g., statins, corticosteroids, CPAP users) excluded or separately analyzed?

Response: We conducted multiple linear regression analysis to identify factors associated with pLL-37. We have described the multiple linear regression analysis in the Results (lines 243–251):

“To identify factors associated with pLL-37, a multivariable regression analysis was conducted using age, %Stage N1, %Stage N3, %Stage REM, arousal index, AHI, ODI, mean SpO₂, minimum SpO₂, and BMI as independent variables, all of which showed significant correlations with pLL-37 in the univariate analysis. The analysis revealed that %Stage N1 (p = 0.040) and AHI (p = 0.038) were independently associated with pLL-37. Because multicollinearity was detected between AHI and ODI, ODI was excluded from the model, and the analysis was repeated. In the revised model, %Stage N1 (p = 0.045) and AHI (p = 0.014) remained significantly associated with pLL-37, suggesting that these variables are independently related to pLL-37 levels.”

a. We did not measure vitamin D levels in this study.

b. We did not adjust for BMI because no correlation was found between BMI and pLL-37. But in response to the reviewer's comment, we additionally included BMI as an independent variable in the multiple linear regression model to adjust for its potential confounding effect. The results were essentially unchanged after adjustment, confirming that BMI was not independently associated with pLL-37.

c. We excluded patients who used any medications known to affect inflammatory markers (such as statins and corticosteroids).

Comment 3. Causality and directionality: Given the cross-sectional observational design, the manuscript should avoid language that implies causality. Phrases suggesting that LL-37 changes "due to" OSA or hypoxic exposure should be revised to reflect the associational nature of the study.

Response: We agree with your opinion and have revised wording to reflect the associational nature of the study.

Comment 4. Biomarker validity: The authors should discuss the pre-analytical variables affecting LL-37 measurement (e.g., hemolysis, freeze-thaw cycles, time from collection to assay). Were samples collected at a standardized time (morning post-PSG)? Diurnal variation of LL-37 has not been well characterized and could introduce variability.

Response: In this study, on the morning following PSG performed for the diagnosis of OSA, we collected blood samples at 06:00, immediately upon the patient’s awakening. We have described the blood collection protocol as follows (lines 141–144):

“On the morning following PSG performed for the diagnosis of OSA, venous blood was drawn from the antecubital region at 06:00, immediately upon the patient’s awakening. Blood was collected using a vacuum tube containing aprotinin. Plasma was immediately separated and cryopreserved at −60 °C until analysis.”

Comment 5. ROC analysis: If the authors present ROC curve data for LL-37 as a diagnostic biomarker for OSA severity, the AUC, sensitivity, specificity, and optimal cut-off values should be clearly reported with 95% confidence intervals. The clinical utility of any proposed cut-off must be discussed in the context of existing OSA diagnosis (PSG remains the gold standard).

Response: To determine the optimal pLL-37 concentration for identifying patients with an AHI > 15, a receiver operating characteristic (ROC) curve was constructed, and the area under the curve (AUC) was calculated. We have described these results at lines 263–272:

“To determine the optimal pLL-37 concentration for identifying patients with an AHI > 15, a receiver operating characteristic (ROC) curve was constructed, and the area under the curve (AUC) was calculated. The AUC was 0.804, indicating good discriminative performance. The maximum value of sensitivity − (1 − specificity) was 0.8. Based on logistic regression analysis, the optimal cut-off value of pLL-37 for diagnosing AHI > 15 was determined to be 56.4 ng/L.

The optimal cut-off value was determined by maximizing the Youden index. At this threshold, the sensitivity, specificity, and Youden index were 85%, 71%, and 0.56, respectively, indicating a moderate-to-good discriminatory ability for identifying patients with AHI >15 events/h.”

Comment 6. Correlation vs. causation in discussion: The discussion should more carefully distinguish between LL-37 as a marker of systemic inflammation secondary to intermittent hypoxia versus a primary mediator of OSA-related pathology. The proposed mechanisms linking LL-37 to sleep architecture disruption are speculative and should be framed accordingly.

Response: We have added the following sentences to the limitations section (lines 342–361):

“In this study, pLL-37 concentrations were independently associated with both the AHI and %Stage N1, a marker of sleep fragmentation and disrupted sleep architecture. However, because of the cross-sectional design of this study, causal relationships cannot be inferred from these associations.

OSA is characterized by intermittent hypoxia, oxidative stress, and systemic inflammation. Elevated pLL-37 levels may therefore reflect these pathophysiological processes and serve as a biomarker of OSA-related inflammatory activity. Conversely, LL-37 has been reported to participate in innate immune regulation, modulation of proinflammatory cytokine production, and tissue remodeling. These biological functions raise the possibility that LL-37 may also contribute to the development and progression of OSA-related pathophysiology. Nevertheless, current evidence is insufficient to determine whether LL-37 acts merely as a marker of disease activity or as a mediator of disease progression.

The association observed between pLL-37 and sleep architecture requires particular caution in interpretation. One possible explanation is that intermittent hypoxia and inflammatory responses associated with OSA promote sleep fragmentation and alterations in sleep architecture, leading to the observed relationship between pLL-37 and %Stage N1 sleep. However, no direct evidence demonstrates that LL-37 influences sleep architecture. Accordingly, our findings should be interpreted as demonstrating an association rather than a causal relationship.”

Minor Concerns:

Comment 1. Figure quality: Ensure all figures meet PLOS ONE resolution requirements (300 DPI minimum) and that axis labels, legends, and statistical annotations are clearly readable.

Response: All figures in this article meet PLOS One resolution requirements (300 DPI minimum).

Comment 2. Tables: Baseline demographic and clinical characteristics (Table 1) should include p-values for between-group comparisons to confirm adequate matching or to justify covariate adjustment.

Response: We have shown the p-values in Table 1.

Comment 3. Reference list: Several foundational references on cathelicidin biology and its role in innate immunity (e.g., Zanetti 2004, Doss et al. 2010) should be cited to provide context for readers unfamiliar with LL-37 biology.

Response: We have added some papers to the reference list.

van Harten RM, van Woudenbergh E, van Dijk A, Haagsman HP. Cathelicidins: immunomodulatory antimicrobials. Vaccines (Basel). 2018;6: 63. doi: 10.3390/vaccines6030063.

Dürr UH, Sudheendra US, Ramamoorthy A. LL-37, the only human member of the cathelicidin family of antimicrobial peptides. Biochim Biophys Acta. 2006;1758: 1408-1425. doi: 10.1016/j.bbamem.2006.03.030

Comment 4. Abbreviations: Ensure all abbreviations are defined at first use and that a consistent abbreviation style is used throughout.

Response: We have double-checked all abbreviations, which are presented in a consistent style.

Comment 5. Limitations section: The limitations section should explicitly acknowledge: (a) cross-sectional design precluding causal inference, (b) potential unmeasured confounders (vitamin D, infections at time of sampling), (c) single-center design limiting generalizability, and (d) the fact that PSG was performed once without accounting for night-to-night variability in sleep architecture.

Response: We have added information to the discussion (lines 362–383).

“Several limitations of this study should be acknowledged. First, the cross-sectional design precludes conclusions regarding causality. Second, this study focused solely on the quantification of circulating pLL-37 concentrations and did not assess the expression of related genes or mRNAs. Therefore, the biological mechanisms underlying the observed associations could not be clarified. Third, blood samples were collected only once in the early morning under fasting conditions. Because LL-37 concentrations may vary according to sampling conditions, including collection time and nutritional status, future studies should compare samples obtained from the same individuals at different timepoints, such as fasting morning and daytime measurements.

In addition, previous studies have reported a positive correlation between salivary LL-37 concentrations and age in children. The present study examined pLL-37 levels exclusively in adults with OSA; therefore, whether similar age-related associations exist in pediatric patients with OSA remains unknown and warrants further investigation.

Finally, LL-37 concentrations may be influenced by unmeasured factors unrelated to OSA, including unidentified comorbidities or other inflammatory conditions. Future longitudinal and mechanistic studies are needed to clarify the causal relationships among LL-37, OSA severity, sleep architecture, and systemic inflammation.

Overall, the available evidence supports a role for pLL-37 as a biomarker of OSA-related inflammation. However, its potential contribution as a biological mediator of OSA pathogenesis remains to be determined.”

Comment 6. CPAP/treatment status: Were any OSA patients already on CPAP therapy? If so, this would significantly confound LL-37 levels, as CPAP treatment reduces systemic inflammation in OSA. This must be clarified.

Response: None of the study participants had received CPAP therapy before the start of the study.

Attachment Submitted filename: Responce_to_Reviewers_PLOS_ONE_LL-37h7N1.doc

# Peer review: Decision Letter 2

**DOI:** 10.1371/journal.pone.0355855.r005

**Authors:** Yongzhong Guo

27 Jul 2026

Plasma levels of antimicrobial peptide LL-37 as a biomarker of sleep quality in patients with obstructive sleep apnea: a clinical observational study

PONE-D-25-66718R2

Dear Dr. Keisuke Hosokawa,

We’re pleased to inform you that your manuscript has been judged scientifically suitable for publication and will be formally accepted for publication once it meets all outstanding technical requirements.

Within one week, you’ll receive an e-mail detailing the required amendments. When these have been addressed, you’ll receive a formal acceptance letter and your manuscript will be scheduled for publication.

An invoice will be generated when your article is formally accepted. Please note, if your institution has a publishing partnership with PLOS and your article meets the relevant criteria, all or part of your publication costs will be covered. Please make sure your user information is up-to-date by logging into Editorial Manager at Editorial Manager® and clicking the ‘Update My Information' link at the top of the page. For questions related to billing, please contact billing support.

If your institution or institutions have a press office, please notify them about your upcoming paper to help maximize its impact. If they’ll be preparing press materials, please inform our press team as soon as possible -- no later than 48 hours after receiving the formal acceptance. Your manuscript will remain under strict press embargo until 2 pm Eastern Time on the date of publication. For more information, please contact onepress@plos.org.

Kind regards,

Yongzhong Guo, Ph.D

Academic Editor

PLOS One

Additional Editor Comments (optional):

Reviewers' comments:

Reviewer's Responses to Questions

**Comments to the Author**

Reviewer #1: All comments have been addressed

Reviewer #2: All comments have been addressed

**********

2. Is the manuscript technically sound, and do the data support the conclusions?

Reviewer #1: Yes

Reviewer #2: Yes

**********

3. Has the statistical analysis been performed appropriately and rigorously?

Reviewer #1: Yes

Reviewer #2: Yes

**********

4. Have the authors made all data underlying the findings in their manuscript fully available?

The PLOS Data policy

Reviewer #1: Yes

Reviewer #2: Yes

**********

5. Is the manuscript presented in an intelligible fashion and written in standard English?

Reviewer #1: Yes

Reviewer #2: Yes

**********

Reviewer #1: (No Response)

Reviewer #2: All the reviewers concerns have been addressed. There is a need for future studies into the role of PLL-37 as a biological mediator in pathogenesis of OSA.

**********

what does this mean?). If published, this will include your full peer review and any attached files.

If you choose “no”, your identity will remain anonymous but your review may still be made public.

**Do you want your identity to be public for this peer review?**  For information about this choice, including consent withdrawal, please see our Privacy Policy

Reviewer #1: No

Reviewer #2: No

**********

# Editor report: Acceptance letter

**DOI:** 10.1371/journal.pone.0355855.r006

**Authors:** Yongzhong Guo

PONE-D-25-66718R2

PLOS One

Dear Dr. Hosokawa,

I'm pleased to inform you that your manuscript has been deemed suitable for publication in PLOS One. Congratulations! Your manuscript is now being handed over to our production team.

At this stage, our production department will prepare your paper for publication. This includes ensuring the following:

* All references, tables, and figures are properly cited

* All relevant supporting information is included in the manuscript submission,

* There are no issues that prevent the paper from being properly typeset

You will receive further instructions from the production team, including instructions on how to review your proof when it is ready. Please keep in mind that we are working through a large volume of accepted articles, so please give us a few days to review your paper and let you know the next and final steps.

Lastly, if your institution or institutions have a press office, please let them know about your upcoming paper now to help maximize its impact. If they'll be preparing press materials, please inform our press team within the next 48 hours. Your manuscript will remain under strict press embargo until 2 pm Eastern Time on the date of publication. For more information, please contact onepress@plos.org.

You will receive an invoice from PLOS for your publication fee after your manuscript has reached the completed accept phase. If you receive an email requesting payment before acceptance or for any other service, this may be a phishing scheme. Learn how to identify phishing emails and protect your accounts at https://explore.plos.org/phishing.

If we can help with anything else, please email us at customercare@plos.org.

Thank you for submitting your work to PLOS ONE and supporting open access.

Kind regards,

PLOS ONE Editorial Office Staff

on behalf of

Dr. Yongzhong Guo

Academic Editor

PLOS One
